# The predictive value of T-cell chimerism for disease relapse after allogeneic hematopoietic stem cell transplantation

**DOI:** 10.3389/fimmu.2024.1382099

**Published:** 2024-04-11

**Authors:** Zhipeng Li, Jing Wang, Lei Deng, Ximin Liu, Fanjun Kong, Yuerong Zhao, Yixi Hou, Fang Zhou

**Affiliations:** Hematology Department, The 960th Hospital of The People’s Liberation Army (PLA) Joint Logistics Support Force, Jinan, China

**Keywords:** chimerism, allo-HSCT, relapse, leukemia, MDS

## Abstract

**Introduction:**

Chimerism is closely correlated with disease relapse after allogeneic hematopoietic stem cell transplantation (allo-HSCT). However, chimerism rate is dynamic changes, and the sensitivity of different chimerism requires further research.

**Methods:**

To investigate the predictive value of distinct chimerism for relapse, we measured bone marrow (BM), peripheral blood (PB), and T-cell (isolated from BM) chimerism in 178 patients after allo-HSCT.

**Results:**

Receiver operating characteristic (ROC) curve showed that T-cell chimerism was more suitable to predict relapse after allo-HSCT compared with PB and BM chimerism. The cutoff value of T-cell chimerism for predicting relapse was 99.45%. Leukemia and myelodysplastic syndrome (MDS) relapse patients’ T-cell chimerism was a gradual decline from 2 months to 9 months after allo-HSCT. Higher risk of relapse and death within 1 year after allo-HSCT. The T-cell chimerism rates in remission and relapse patients were 99.43% and 94.28% at 3 months after allo-HSCT (*P* = 0.009), 99.31% and 95.27% at 6 months after allo-HSCT (*P* = 0.013), and 99.26% and 91.32% at 9 months after allo-HSCT (*P* = 0.024), respectively. There was a significant difference (*P* = 0.036) for T-cell chimerism between early relapse (relapse within 9 months after allo-HSCT) and late relapse (relapse after 9 months after allo-HSCT) at 2 months after allo-HSCT. Every 1% increase in T-cell chimerism, the hazard ratio for disease relapse was 0.967 (95% CI: 0.948–0.987, *P*<0.001).

**Discussion:**

We recommend constant monitoring T-cell chimerism at 2, 3, 6, and 9 months after allo-HSCT to predict relapse.

## Introduction

Allogeneic hematopoietic stem cell transplantation (allo-HSCT) provides the most effective therapy in hematological malignancies (HM) and is used to treat leukemia and myelodysplasia (MDS) (1, 2). Despite the enormous benefit of allo-HSCT and improved patient overall survival, disease relapse remains the primary obstacle to successful allo-HSCT. Although the majority of patients with leukemia/MDS can be cured by allo-HSCT, almost all high-risk patients will eventually relapse (3). Relapse represents the leading cause of failure in patients after allo-HSCT and the clinical prognosis for HM patients with allo-HSCT failure is poor (4). Relapse is the most frequent cause of death in transplant patients (5). Notably, deaths due to relapse account for nearly 30% of all deaths within 100 days post-transplant (6). As a consequence, an ideal strategy would be to monitor patients who are at risk of relapses early and then institute effective therapies.

Multiple studies have demonstrated that chimerism monitoring might be associated with HM relapse after allo-HSCT (6, 7). Chimerism can broadly be defined as being able to quantify donor and recipient hematopoiesis and can be conducted in T cells, peripheral blood (PB) and bone marrow (BM) (6, 8, 9). The complete donor chimerism relies on allo-HSCT and the complete reconstruction immune system in HM patients (10). Generally, posttransplant chimerism is a dynamic phenomenon and the mixed chimerism (MC) may be indicative of relapse (11). Given the relapse and poor outcomes in the disease of leukemia/MDS, it is necessary to verify the predictive value of donor chimerism. In the present study, we compare and contrast the dynamic range difference between T-cell chimerism, PB chimerism, and BM chimerism and put forward the most appropriate testing method.

## Methods

### Patients

After approval of the Ethical Committee of the 960th Hospital of the People’s Liberation Army, a cohort of 178 allo-HSCT patients who underwent chimerism determination was retrospectively analyzed. Chimerism determination was performed at least twice.

### Evaluation of donor-host chimerism

#### Magnetic bead sorting

Magnetic bead sorting separates T cells by binding antibodies on magnetic beads to antigens on T cell surfaces. BM is collected in a centrifuge tube, an appropriate amount of antibody is added and shaken well, followed by the addition of pre-prepared magnetic beads, shaken again, and allowed to stand. Place the tube in the magnetic pole, and the magnetic beads will adhere to the tube’s wall, removing the unbound cell solution while binding the T cells to the magnetic beads. PBS was added and washed twice, resulting in purified T cells with over 90% purity.

#### STR-PCR

DNA was extracted from PB/BM/purified T cells and amplified for 18 STR markers, including THO1, D21S11, D2S1338, Penta E, D5S818, D13S317, D7S820, D16S539, CSF1PO, Amel, VWA, D8S1179, TPOX, FGA, D6S1043, D12S391, D10S1248, and Penta D. STR-PCR conditions: pre-denaturation at 95°C for 660s, denaturation at 94°C for 20s, annealing at 59°C for 120s, extension at 72°C for 60s, 35 cycles total, and final extension at 60°C for 1500s. The peak graphs were examined to determine the results of STR typing for the reference products.

### Definitions

HM relapse was defined by the reappearance of leukemic blasts in PB or relapse of BM blasts ≥5% or the development of extramedullary disease after allo-HSCT (12, 13). T-cell, PB, and BM donor chimerism were monitored at different time points after transplantation. Complete chimerism (CC) was broadly defined as >95% donor cells in the PB and BM samples and MC was defined as 5 to 95% donor cells (12, 14).

### Statistical analysis

Descriptive statistics were used to summarize the clinical characteristics of patients in the study. The quantitative data were expressed as mean ± SD (standard deviation). Shapiro–Wilk and Levene test were used to analyze normality and homogeneity of variance of all experimental data. Data with normally and homogeneously distributed were analyzed using SPSS 26.0 by one-way analysis of variance (ANOVA). When all variables in the distribution failed to meet the assumptions of normality and homogeneity, Kruskal-Wallis one-way ANOVA by ranks was used for data evaluation. Significant differences were defined as *p*-values less than 0.05. Diagnostic accuracy of chimerism on relapse was compared using receiver operating characteristic (ROC) curves. The influence of chimerism at different periods of relapse was evaluated using the Cox proportional hazards model.

## Results

### Patients

Between May 2017 and June 2023, 178 patients underwent allo-HSCT for leukemia/MDS in our institution. In case of multiple relapse, only the first relapse was considered. One hundred fifty-nine patients underwent T-cell chimerism testing. In addition, 170 patients underwent BM chimerism testing, while 144 underwent PB chimerism testing. Baseline characteristics of patients are detailed in **Table 1**.

**Table 1:** Baseline characteristics of patients in chimerism analyses.

	Donor chimerism		
	T-cell chimerism	BM chimerism	PB chimerism
Patients	159	170	144
Age at transplant, years (median;range)	38 (18–66)	38 (18–66)	38 (18–66)
Sex			
Female	78	84	75
Male	81	86	69
Underlying disease			
Leukemia	127	134	109
MDS	32	36	35
Donor type			
HID, *n* (%)	95	101	77
MSD, *n* (%)	53	57	56
URD, *n* (%)	11	12	11
Stem cell source			
Peripheral blood	119	126	105
Bone marrow	1	1	1
Peripheral blood+Bone marrow	39	43	38
Infused cells			
MNC (×10^8^/kg)	10.68 (1.64–32.75)	10.67 (1.64–32.75)	10.6 (1.64–24.26)
CD34+ cells (×106/kg)	3.73 (0.024–22.08)	3.64 (0.024–22.08)	3.4 (0.011–12.232)
Conditioning regimen			
TBI 1000; CY, IDA based (with or without ATG, Ara-c, TT or Azacitidine)	2	2	2
TBI 300–400; CY based (with or without Ara-c, ATG, Flu, MIT, Meccnu or IDA)	36	36	24
No TBI; BU based (with or without CY, FLU, Ara-c, ATG, DAC, Melphalan, Meccnu)	121	132	118
aGvHD prophylaxis			
CsA + MMF+sMTX (with or without CY, or FK506)	149	160	137
CsA + sMTX (with or without CY or FK506)	10	10	7

BM, bone marrow; PB, peripheral blood; MDS, myelodysplastic syndrome; HID, haploidentical donor; MSD, matched sibling donor; URD, unrelated donor; MNC, mononuclear cells; TBI, total body irradiation (in cGy); CY, cyclophosphamide; IDA, Idarubicin; ATG, antithymocyte globulin; Ara-C, Cytarabine; TT, Thiotepa; Flu, fludarabine; MIT, Mitoxantrone Liposomes; BU, busulfan; DAC, Decitabine; GVHD, graft-versus-host disease; CsA, cyclosporine; sMTX, short-term methotrexate.

### The number of patients and chimerism status

**Figure 1:**
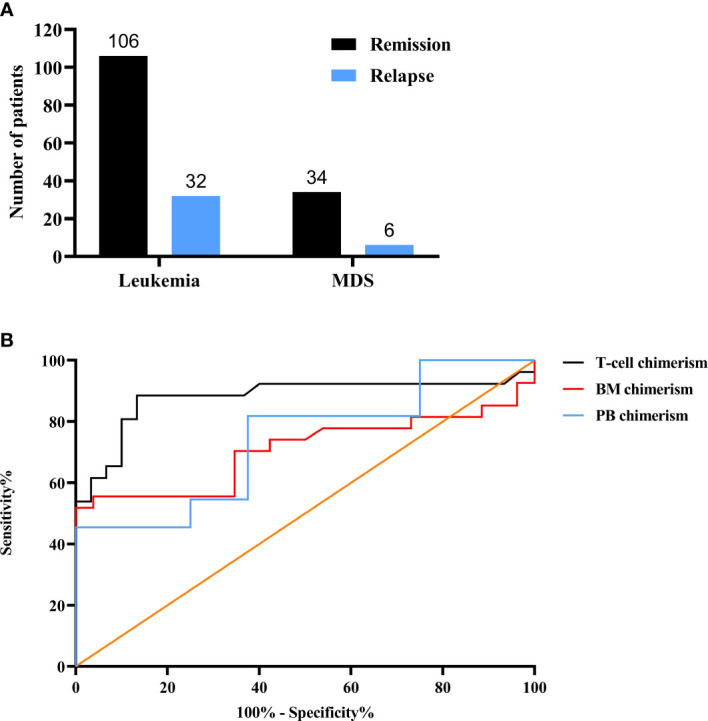
Chimerism status after allo-HSCT. **(A)** The numbers of leukemia and MDS. **(B)** The comparison of three different chimerism using ROC curves. MDS, myelodysplasia; ROC, receiver operating characteristic; BM, bone marrow; PB, Peripheral blood.

In total, 178 leukemia/MDS allo-HSCT patients were entered into the study cohort. The total number of remission in leukemia/MDS was 106 and 34, respectively, whereas the number of relapses was 32 and 6 (**Figure 1A**). In the ROC curve, T-cell chimerism showed the highest area under the curve (AUC) value (**Figure 1B**). The cutoff value of the T-cell chimerism was 99.45%, the sensitivity was 88.5%, and the specificity was 86.7% (AUC = 0.879, *P* < 0.001). The cutoff value of the BM chimerism was 98.12%, the sensitivity was 51.9%, and the specificity was 100% (AUC = 0.707, *P* = 0.01). The cutoff value of the PB chimerism was 98.18%, the sensitivity was 45.5%, and the specificity was 100% (AUC = 0.739, *P* = 0.083).

### Comparison of donor-host chimerism in relapse patients at different time intervals after allo-HSCT

**Figure 2:**
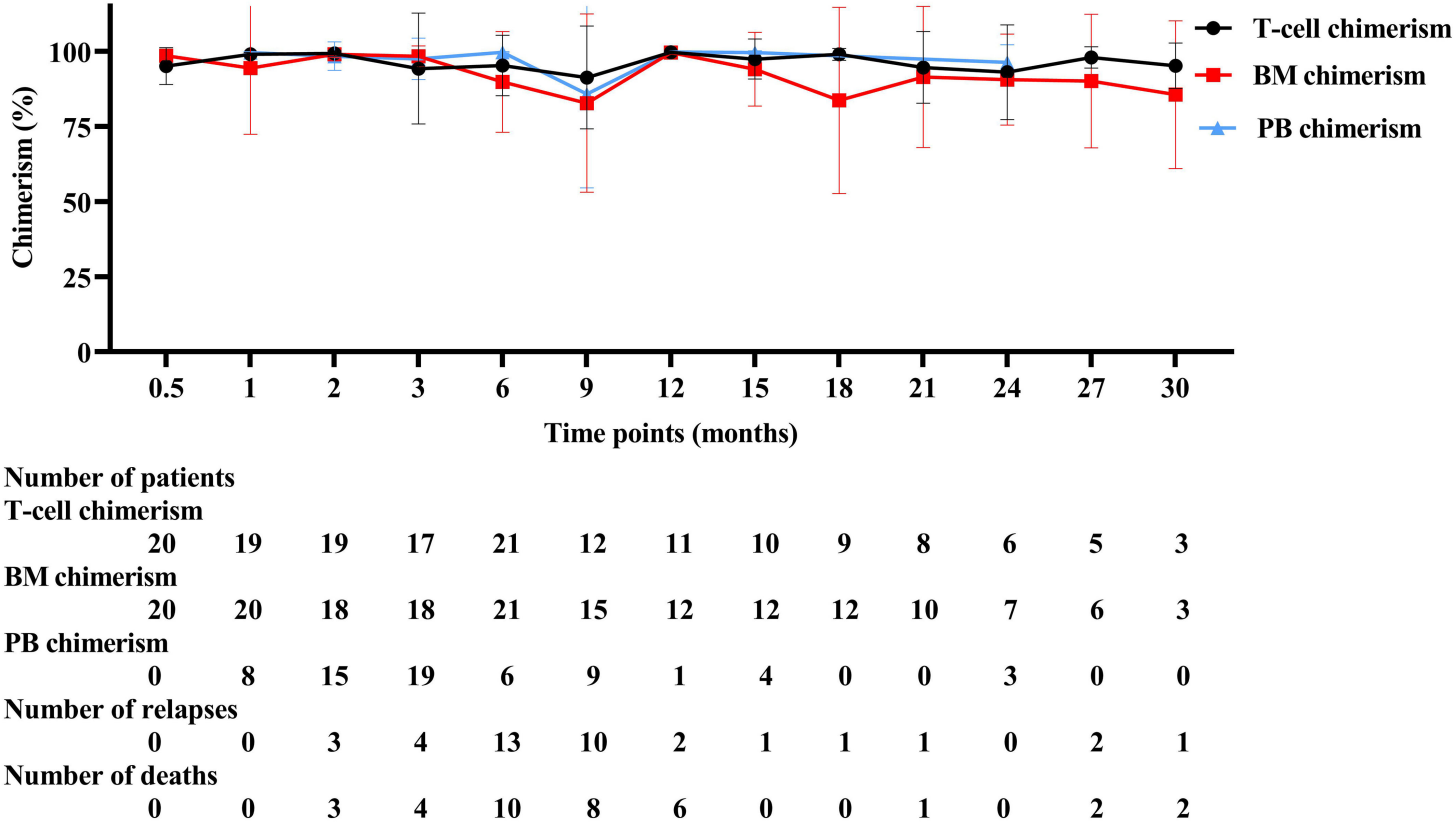
Three different chimerism in relapse patients. BM, bone marrow, PB, peripheral blood.

There was a high risk of relapse and mortality in relapsed patients within 12 months after allo-HSCT and three chimerism displayed a downward trend as a whole (**Figure 2**). Chimerism reached its highest point at 12 months after allo-HSCT. T-cell chimerism was statistically significant for 9 months and 12 months after allo-HSCT (*P* = 0.001). Detailed values are shown in **Supplementary Table S1**.

### Comparison of donor-host chimerism in remission and relapse patients at different time intervals after allo-HSCT

**Figure 3:**
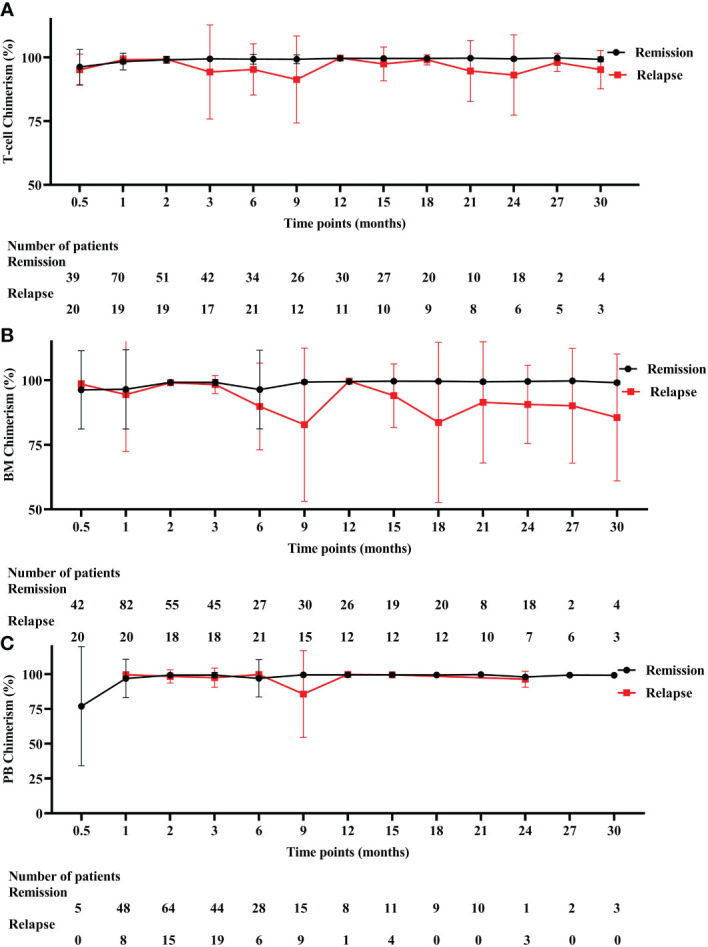
Three different chimerism in remission and relapsed patients. **(A)** T-cell chimerism. **(B)** BM chimerism. **(C)** PB chimerism. BM, bone marrow, PB, peripheral blood.

T-cell chimerism decreased progressively from 2 months after allo-HSCT and dropped to the lowest point at 9 months (**Figure 3A**). T-cell chimerism of remission and relapsed patients at 3 months was 99.43% and 94.28% (*P* = 0.009), respectively. T-cell chimerism of remission and relapsed patients at 6 months was 99.31% and 95.27% (*P* = 0.013), respectively. T-cell chimerism of remission and relapsed patients at 9 months was 99.26% and 91.32% (*P* = 0.024), respectively. BM chimerism decreased progressively from 3 months after allo-HSCT and dropped to the lowest point at 9 months (**Figure 3B**). BM chimerism of remission and relapsed patients at 6 months was 96.38% and 89.82% (*P* = 0.044), respectively. BM chimerism of remission and relapsed patients at 9 months was 99.33% and 82.79% (*P* = 0.198), respectively. PB chimerism decreased progressively from 6 months after allo-HSCT and dropped to the lowest point at 9 months (**Figure 3C**). PB chimerism of remission and relapsed patients at 9 months was 99.58% and 85.78% (*P* = 0.531), respectively. Detailed values are shown in **Supplementary Table S2**.

### Comparison of donor-host chimerism in early relapse and late relapse patients at different time intervals after allo-HSCT

**Figure 4:**
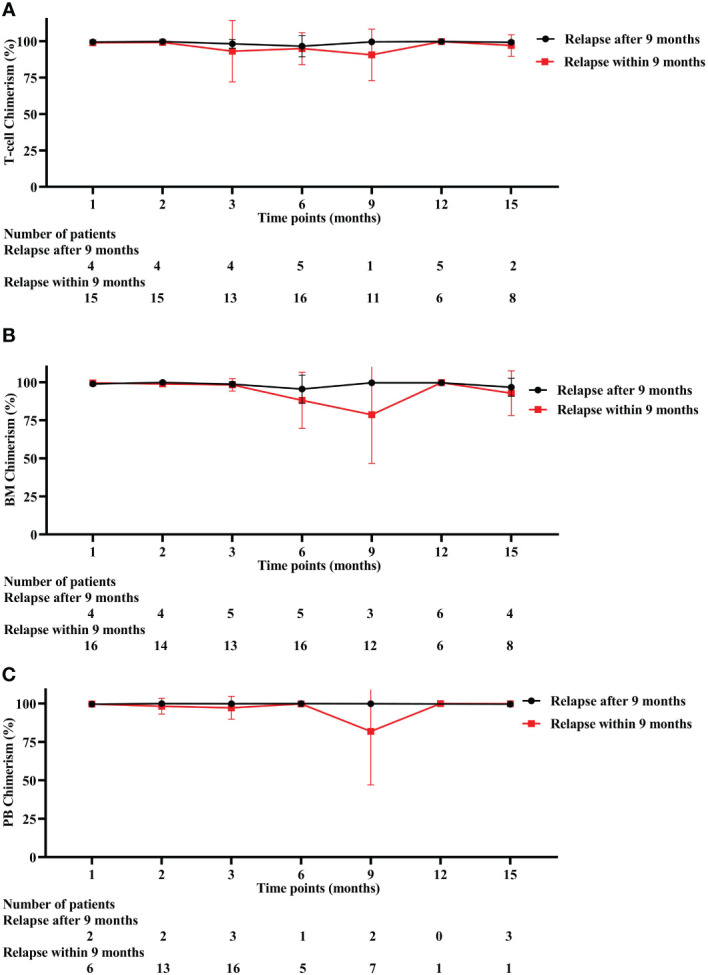
Three different chimerism in early and late relapse patients. **(A)** T-cell chimerism. **(B)** BM chimerism. **(C)** PB chimerism. BM, bone marrow, PB, peripheral blood.

Among 38 relapse patients, T-cell chimerism of early relapse (relapse within 9 months after allo-HSCT) and late relapse (relapse after 9 months after allo-HSCT) was 99.16% and 99.76% (*P* = 0.036) at 2 months after allo-HSCT, respectively (**Figure 4A**). BM chimerism of early relapse patients showed a decreasing trend at 3 months after allo-HSCT, while the PB chimerism decreasing tendency was preserved at 6 months after allo-HSCT (**Figures 4B, C**). Detailed values are shown in **Supplementary Table S3**.

### Relapse risk

We created a univariate Cox model for T-cell chimerism predicting relapse.

T-cell chimerism showed that compared with the CC, MC had increased odds of relapse (Hazard Ratio = 5.377, 95% CI: 2.249–12.853, *P* < 0.001). In addition, every 1% increase in T-cell chimerism, the hazard ratio for disease relapse was 0.967 (95% CI: 0.948–0.987, *P* < 0.001).

## Discussion

Due to failure to avoid relapse of disease, relapse patients after allo-HSCT had a poor prognosis and limited therapies (15). HM relapse remains the primary cause of the dismal prognosis after allo-HSCT (16, 17). Thus, precision predicting the relapse of disease after allo-HSCT to guide treatment decisions is particularly important. To predict the relapse of high-risk individuals early and take specific intervention measures in time, it is helpful for clinicians to reduce the poor outcomes of patients. Related studies have confirmed that the chimerism status was strongly associated with the risk of disease relapse (7, 18–20). Chimerism is a dynamic process and ever-changing, and MC is responsible for allo-HSCT failure (21). The most appropriate chimerism analysis is based on disease and allo-HSCT type (22). However, there is a lack of comparative analysis of different chimerism of dynamic changes. This report details the evolution of dynamic changes in T-cell chimerism, BM chimerism, and PB chimerism at different points after allo-HSCT. By examining these chimerism statuses, one can identify the chimerism that is most strongly linked to relapse, which offers valuable reference information for clinical practice.

The ROC curve is universally used as a method for assessing the accuracy of potential biomarkers by calculating the area under the ROC curve (AUC) (23–25). As shown in the ROC curve, compared with BM chimerism and PB chimerism, T-cell chimerism had the highest AUC and was more suitable for the prediction of disease relapse. Through successive tests can be able to effectively identify high-risk relapse patients, which indicates close monitoring is extremely important (26, 27). Donor cells need time to rebuild the hematopoietic system after allo-HSCT, so it is not advisable to assess the chimerism status after transplantation too early (22). In this study, the study was examined from 15 days after allo-HSCT, and the study’s duration was restricted to a 30-month follow-up period. We found that the number of relapse and death patients is continuously increasing with a decrease in chimerism rate from 2 to 9 months after allo-HSCT. The change trend of the chimerism rate is opposite to the number of relapse and death patients. The relapse of leukemia/MDS is accompanied by a drop in donor chimerism (22). Hence, these findings further support that chimerism can predict clinical relapse. Given the decrease in the number of relapsed patients after 9 months, these findings imply that 9 months following allo-HSCT may be an important turning point.

For those with relapse patients, high-risk relapse mainly occurs in 12 months after allo-HSCT. A relatively large of relapse and death was at this stage. Early relapse affects the state of T-cell chimerism after allo-HSCT. Compared with late relapse patients, early relapse patients’s T-cell chimerism rate decreased gradually from 2 months to 9 months after allo-HSCT in 38 relapse samples. Nine months after allo-HSCT as an important time point, the T-cell chimerism rate is transformed from low to high. Changes in T-cell chimerism were most pronounced at 3, 6, and 9 months after allo-HSCT in patients with early relapses, which can serve as a preventive screen for disease relapse earlier, whereas 2 months failed to detect a difference in remission and relapse patients, perhaps related to the small sample size.

It is notable that we found MC with a high probability of relapse by univariate Cox regression analysis. The relapse incidence of mixed T-cell chimerism was several times higher than complete donor chimerism. Mixed T-cell chimerism requires close monitoring and additional interventions due to its high risk of relapse. With each 1% increase in the T-cell chimerism, the probability of relapse of leukemia/MDS after allo-HSCT decreases by 3.3%.

Furthermore, as an independent predictor, MRD detection has a strong clinical correlation with disease relapse after allo-HSCT, which is useful for guiding treatment decisions. With the use of NGS and other technologies, MRD detection becomes more sensitive, overcoming the low sensitivity of chimerism in STR-PCR detection. When there are no high-specificity immunophenotypic markers, chimerism detection can be used to supplement them. The feasibility of combining the two in clinical research has been confirmed in related studies.

In conclusion, our research clarifies that, in contrast to BM and PB chimerism, continuous T-cell chimerism monitoring predicting relapse is more accurate. Therefore, we recommend that the T-cell chimerism rate should be closely monitored for 2, 3, 6, and 9 months after allo-HSCT. This would be helpful for early detection of relapse after allo-HSCT and timely interventions.

## Data availability statement

The raw data supporting the conclusions of this article will be made available by the authors, without undue reservation.

## Ethics statement

The studies involving humans were approved by The Ethical Committee of the 960th Hospital of the People’s Liberation Army. The studies were conducted in accordance with the local legislation and institutional requirements. The human samples used in this study were acquired from primarily isolated as part of your previous study for which ethical approval was obtained. Written informed consent for participation was not required from the participants or the participants’ legal guardians/next of kin in accordance with the national legislation and institutional requirements.

## Author contributions

ZL: Writing – original draft. JW: Writing – original draft. LD: Conceptualization, Writing – original draft. XL: Data curation, Writing – original draft. FK: Methodology, Writing – original draft. YZ: Formal analysis, Writing – review & editing. YH: Software, Writing – review & editing. FZ: Writing – review & editing.
