# Supplementary material for: The predictive value of T-cell chimerism for disease relapse after allogeneic hematopoietic stem cell transplantation
Source: Front Immunol. 2024 Apr 11;15:1382099. doi: 10.3389/fimmu.2024.1382099 (PMC11043518; doi:10.3389/fimmu.2024.1382099)
Supplement: Supplementary file 1 [file Table_1.docx]

**Supplementary Table 1**

**The median value and range of three different chimerism in relapse patients**

|  | **Donor Chimerism (relapse patients)** | | |
| --- | --- | --- | --- |
| **Time points (months)** | **T-cell chimerism** | **BM chimerism** | **PB chimerism** |
| 0.5 | 97.46%(78.18%-99.75%) | 98.85%(95.25%-99.85%) | — |
| 1 | 99.53%(92.99%-99.99%) | 99.7%(96.68%-99.99%) | 99.63%(99.16%-99.93%) |
| 2 | 99.62%(95.83%-99.85%) | 99.42%(95.53%-99.91%) | 99.77%(81.31%-99.97%) |
| 3 | 99.18%(22.94%-99.91%) | 99.37%(84.79%-99.9%) | 99.78%(72.76%-99.96%) |
| 6 | 99.4%(57.73%-99.91%) | 98.73%(49.14%-99.91%) | 99.75%(99.51%-99.97%) |
| 9 | 99.3%(43.21%-99.72%) | 99.32%(10.23%-99.93%) | 99.57%(7.26%-100%) |
| 12 | 99.77%(99.37%-99.96%) | 99.63%(99%-99.94%) | 99.84% |
| 15 | 99.5%(78.58%-99.94%) | 99.42%(58.31%-99.99%) | 99.84%(98.76%-99.94%) |
| 18 | 99.78%(93.82%-99.87%) | 99.55%(7.52%-99.82%) | — |
| 21 | 99.11%(65.24%-99.92%) | 99.51%(24.74%-99.96%) | — |
| 24 | 99.54%(60.95%-99.93%) | 99.21%(64%-99.91%) | 99.64%(89.74%-99.84%) |
| 27 | 99.73%(91.62%-99.8%) | 99.18%(44.76%-99.79%) | — |
| 30 | 99.12%(86.53%-100%) | 99.54%(57.25%-99.97%) | — |
