# Supplementary material for: The predictive value of T-cell chimerism for disease relapse after allogeneic hematopoietic stem cell transplantation
Source: Front Immunol. 2024 Apr 11;15:1382099. doi: 10.3389/fimmu.2024.1382099 (PMC11043518; doi:10.3389/fimmu.2024.1382099)
Supplement: Supplementary file 2 [file Table_2.docx]

**Supplementary Table 2**

**The median value and range of three different chimerism in remission and relapse patients**

|  | **Donor Chimerism (remission/relapse patients)** | | |
| --- | --- | --- | --- |
| **Time points (months)** | **T-cell chimerism** | **BM chimerism** | **PB chimerism** |
| 0.5 | 98.94%(67.19%-99.83%)/  97.46%(78.18%-99.75%) | 99.22%(91.31%-99.97%)/  98.85%(95.25%-99.85%) | 98.67%(86.5%-99.64%)/  — |
| 1 | 99.47%(82.56%-99.96%)/  99.53%(92.99%-99.99%) | 99.54%(84.36%-99.98%)/  99.7%(96.68%-99.99%) | 99.63%(4.24%-99.95%)/  99.63%(99.16%-99.93%) |
| 2 | 99.45%(92.05%-99.97%)/  99.62%(95.83%-99.85%) | 99.64%(93.11%-100%)/  99.42%(95.53%-99.91%) | 99.65%(91.99%-99.97%)/  99.77%(81.31%-99.97%) |
| 3 | 99.64%(97.38%-100%)/  99.18%(22.94%-99.91%) | 99.62%(93.47%-99.94%)/  99.37%(84.79%-99.9%) | 99.69%(87.53%-99.96%)/  99.78%(72.76%-99.96%) |
| 6 | 99.7%(88.59%-100%)/  99.4%(57.73%-99.91%) | 99.61%(20.39%-99.93)/  98.73%(49.14%-99.91%) | 99.7%(99.69%-99.71%)/  99.75%(99.51%-99.97%) |
| 9 | 99.61%(90.94%-99.93%)/  99.3%(43.21%-99.72%) | 99.61%(96.64%-99.93%)/  99.32%(10.23%-99.93%) | 99.67%(98.41%-99.96%)/  99.57%(7.26%-100%) |
| 12 | 99.74%(98.87%-99.92%)/  99.77%(99.37%-99.96%) | 99.55%(98.88%-99.92%)/  99.63%(99%-99.94%) | 99.74%(98.37%-99.87%)/  99.84% |
| 15 | 99.67%(98.58%-99.96%)/  99.5%(78.58%-99.94%) | 99.64%(98.82%-99.93%)/  99.42%(58.31%-99.99%) | 99.66%(98.2%-99.93%)/  99.84%(98.76%-99.94%) |
| 18 | 99.64%(98.64%-99.98%)/  99.78%(93.82%-99.87%) | 99.64%(98.64%-99.98%)/  99.55%(7.52%-99.82%) | 99.53%(98.42%-99.92%)/  — |
| 21 | 99.68%(99.35%-99.91%)/  99.11%(65.24%-99.92%) | 99.6%(97.97%-99.86%)/  99.51%(24.74%-99.96%) | 99.84%(98.65%-99.94%)/  — |
| 24 | 99.70%(96.75%-99.91%)/  99.54%(60.95%-99.93%) | 99.64%(99%-99.95%)/  99.21%(64%-99.91%) | 98.09%/  99.64%(89.74%-99.84%) |
| 27 | 99.83%(99.8%-99.85%)/  99.73%(91.62%-99.8%) | 99.76%(99.68%-99.83%)/  99.18%(44.76%-99.79%) | 99.3%(98.81%-99.79%)/  — |
| 30 | 99.61%(97.58%-99.87%)/  99.12%(86.53%-100%) | 99.16%(97.91%-99.76%)/  99.54%(57.25%-99.97%) | 99.31%(98.84%-99.54%)/  — |
