# Supplementary material for: The predictive value of T-cell chimerism for disease relapse after allogeneic hematopoietic stem cell transplantation
Source: Front Immunol. 2024 Apr 11;15:1382099. doi: 10.3389/fimmu.2024.1382099 (PMC11043518; doi:10.3389/fimmu.2024.1382099)
Supplement: Supplementary file 3 [file Table_3.docx]

**Supplementary Table 3**

**The median value and range of three different chimerism in early relapse and late relapse patients**

|  | **Donor Chimerism (early/late relapse patients)** | | |
| --- | --- | --- | --- |
| **Time points (months)** | **T-cell chimerism** | **BM chimerism** | **PB chimerism** |
| 1 | 99.61%(92.99%-99.9%)/  99.25%(98.67%-99.83%) | 99.69%(98.16%-99.99%)/  99.43%(96.68%-99.89%) | 99.63%(99.33%-99.93%)/  99.55%(99.16%-99.93%) |
| 2 | 99.59%(95.83%-99.79%)/  99.75%(99.65%-99.85%) | 99.31%(95.53%-99.91%)/  99.79%(99.59%-99.91%) | 99.73%(81.31%-99.93%)/  99.89%(99.81%-99.97%) |
| 3 | 98.91%(96.61%-99.81%)/  96.73%(93.67%-99.79%) | 99.35%(84.79%-99.88%)/  99.39%(95.71%-99.9%) | 99.78%(72.76%-99.96%)/  99.84%(99.61%-99.84%) |
| 6 | 99.28%(94.03%-99.78%)/  99.59%(83.64%-99.91%) | 96.57%(49.14%-99.9%)/  99.7%(79.02%-99.91%) | 99.65%(99.51%-99.97%)/  99.84% |
| 9 | 99.22%(43.21%-99.72%)/  99.49% | 99.23%(10.23%-99.93%)/  99.68%(99.32%-99.9%) | 99.51%(7.26%-99.92%)/  99.83%(99.65%-100%) |
| 12 | 99.78%(99.37%-99.83%)/  99.66%(99.64%-99.89%) | 99.63%(99.43%-99.88%)/  99.7%(99%-99.94%) | 99.84%/  — |
| 15 | 99.54%(99.36%-99.9%)/  99.1%(98.54%-100%) | 99.42%(58.31%-99.97%)/  99.44%(87.92%-99.99%) | 99.8%/  99.87%(98.76%-99.94%) |
